# Editorial: Specialized metabolites manipulating organismal behaviors and rhizospheric communications

**DOI:** 10.3389/fpls.2023.1197058

**Published:** 2023-04-20

**Authors:** Jian You Wang, Valentina Fiorilli, Luisa Lanfranco, Tadao Asami, Salim Al-Babili

**Affiliations:** ^1^ The BioActives Lab, Center for Desert Agriculture, King Abdullah University of Science and Technology, Thuwal, Saudi Arabia; ^2^ Department of Life Sciences and Systems Biology, University of Torino, Torino, Italy; ^3^ Graduate School of Agricultural and Life Sciences, The University of Tokyo, Tokyo, Japan; ^4^ The Plant Science Program, Biological and Environmental Science and Engineering Division, King Abdullah University of Science and Technology (KAUST), Thuwal, Saudi Arabia

**Keywords:** root exudates, plant secondary metabolites, plant-plant interaction, plant microbe communication, phytohormones, rhizospheric communication

Evolving from aqueous green algae, sessile terrestrial plants have developed diverse mechanisms to overcome challenging environments and cope with different types of stress (Weng et al., 2021). For a better survival, plants utilize chemical signals that affect and are perceived by surrounding organisms. Many of these compounds are released by roots into the soil and act as signaling molecules in the communication within the rhizosphere. Indeed, plants leak out a complex cocktail of chemical signals mediating plant-plant, plant-bacteria, and plant-fungi interactions (Massalha et al., 2017). Many of these specialized signals derive from secondary metabolic pathways. For example, Ke et al. summarized that the breakdown of carotenoids provides (i) the precursor for apocarotenoid signaling and regulatory metabolites (Dickinson et al., 2019; Jia et al., 2019; Wang et al., 2019; Votta et al., 2022; Ablazov et al.,2023), and (ii) for the plant hormones abscisic acid and strigolactones (SLs). Both hormones are important for plant growth and development, and response to biotic and abiotic stress stimuli (Al-Babili and Bouwmeester, 2015; Wang et al., 2021). Moreover, SLs are some of the best-known examples of underground signaling molecules in plant-microbe and plant-plant communication (Lanfranco et al., 2018). In their study, Wang et al. proposed that root-released SLs function as an important rhizospheric signaling cue, more specifically canonical SLs (Ito et al., 2022; Wheeldon et al., 2022; Yoneyama et al., 2022). However, they are not the only ones shaping underground communications; many other intriguing root-released metabolites, such as 6-methoxy-2-benzoxazolin, blumenols, and camalexin, are also involved in the rhizospheric interactions (Hu et al., 2018; Fiorilli et al.; Koprivova et al., 2023).

Despite the progress that has been made in the past decades, the biological role of root exudates in shaping organismal communications and interactions remained largely elusive. Research in this field has been aggravated by the very low quantities of root-secreted specialized metabolites, which hinders their identification, structural characterizations, and detailed assessment of their biological function. Therefore, researchers need to collect root exudates at large scale to get insights into their metabolite compositions (Ueno et al., 2021). Indeed, the improvement of root exudate collection and concentration techniques allowed the identification and structural characterization of new compounds such as (i) three novel canonical SLs in tomato reported by Wakabayashi et al., which might pave the way to the identification of solanacol biosynthesis, and (ii) the confirmation by Oota et al. that pectic carbohydrates released *by Lotus corniculatus L.* are important nematode attractants regulating (micro)organismal chemotaxis in the rhizosphere. The latter role was discovered using the super-growing root culture system, providing a promising tool for root pathogen research. Although several analytical protocols have been proposed, developed, and employed (Van Dam and Bouwmeester, 2016; Escolà Casas and Matamoros, 2021; Wang et al., 2022), Salem et al. discussed and proposed that a comprehensive analysis of rhizosphere metabolome is still challenging in chemical ecology research.

In summary, this Research Topic offers updated knowledge about allelopathic, rhizospheric secondary metabolites in plant-plant, -animal, and -microbe communications. The information presented can be helpful in future developments towards increasing crop performance and decreasing the ecological and economic loss for sustainable agriculture; for instance, several analogs/mimics of these specialized metabolites have been designed and utilized in basic research as well as in agricultural applications (Rigal et al.; Vaidya et al., 2019; Jamil et al.; Wang et al., 2020; Jamil et al., 2022; Wang et al.). Finally, our research collection also provides important perspectives on the overlooked regulatory and signaling metabolites in the rhizosphere, paving the way for future investigations.

## Author contributions

All authors have made a substantial, direct, and intellectual contribution to the work, and approved it for publication.
